# Zero-premium Medicare Advantage plans: trends in areas with socioeconomic vulnerability and health needs

**DOI:** 10.1093/haschl/qxaf177

**Published:** 2025-09-19

**Authors:** Changchuan Jiang, Lesi He, Chuan Angel Lu, Arthur S Hong, Xin Hu, Joseph H Joo, Ryan D Nipp, Ya-Chen Tina Shih, K Robin Yabroff, Joshua M Liao

**Affiliations:** Division of Hematology and Oncology, Department of Internal Medicine, University of Texas Southwestern Medical Center, Dallas, TX 75390, United States; Peter O'Donnell Jr. School of Public Health, University of Texas Southwestern Medical Center, Dallas, TX 75390, United States; Program on Policy Evaluation and Learning, Dallas, TX 75390, United States; Department of Health Data Science and Biostatistics, Peter O’Donnell Jr. School of Public Health, University of Texas Southwestern Medical Center, Dallas, TX 75390, United States; Division of Hematology and Oncology, Department of Internal Medicine, University of Texas Southwestern Medical Center, Dallas, TX 75390, United States; Peter O'Donnell Jr. School of Public Health, University of Texas Southwestern Medical Center, Dallas, TX 75390, United States; Program on Policy Evaluation and Learning, Dallas, TX 75390, United States; Division of General Internal Medicine, Department of Internal Medicine, University of Texas Southwestern Medical Center, Dallas, TX 75390, United States; Department of Radiation Oncology, Emory University School of Medicine, Atlanta, GA 30322, United States; Division of General Internal Medicine, Department of Internal Medicine, University of Texas Southwestern Medical Center, Dallas, TX 75390, United States; Department of Medicine, University of Oklahoma Health Sciences Center, Oklahoma City, OK 73104, United States; Program in Cancer Health Economics Research, Jonsson Comprehensive Cancer Center, and Department of Radiation Oncology, School of Medicine, University of California, Los Angeles, CA 90095, United States; Surveillance and Health Equity Science, American Cancer Society, Atlanta, GA 30303, United States; Peter O'Donnell Jr. School of Public Health, University of Texas Southwestern Medical Center, Dallas, TX 75390, United States; Program on Policy Evaluation and Learning, Dallas, TX 75390, United States; Division of General Internal Medicine, Department of Internal Medicine, University of Texas Southwestern Medical Center, Dallas, TX 75390, United States

**Keywords:** Medicare Advantage, MA, zero-premium plans, enrollment trends, access to care, health equity, plan performance, plan star ratings, quality of care, socioeconomic disadvantage, vulnerable populations

## Abstract

**Introduction:**

Zero-premium Medicare Advantage (MA) plans are increasingly popular, yet knowledge gaps exist regarding their distribution, enrollment, and quality, particularly in areas with greater socioeconomic vulnerability and clinical need.

**Methods:**

We conducted a serial cross-sectional study of publicly available CMS data from 2019-2024, analyzing 2472 US counties. Annual plan counts and enrollment rates were examined, stratified by county-level socioeconomic and health characteristics (racial/ethnic minority percentage, poverty rate, and prevalence of fair/poor health). Counties were categorized into quartiles for comparison.

**Results:**

Zero-premium MA plans expanded substantially from 2019-2024, rising from 46.02% of MA plans (9.12 million enrollees) to 66.3% (18.76 million). These plans were more likely to feature restrictive provider networks and showed disproportionate enrollment growth in counties with greater socioeconomic and health needs (higher proportions of racial/ethnic minority residents, poverty, and poor health status; *P* < 0.001). Across all county-characteristic subgroups, zero-premium plans consistently had lower star ratings (1-3.5).

**Conclusion:**

Rapid zero-premium MA plan adoption raises concerns about the quality of care, especially among vulnerable populations. Further examination of plan quality standards and patient outcomes, transparency of enrollment incentives (eg, insurance broker commissions), and enrollee navigation and decision-making about plan options is warranted.

## Teaser

Does the growing popularity of zero-premium Medicare Advantage (MA) plans reflect a secure option for beneficiaries, balancing broad availability with consistent quality of service? Offering seemingly “free” coverage, these plans are rapidly gaining market share, especially in communities with significant socioeconomic and health needs. This study uncovers a complex picture: Zero-premium plans are indeed becoming more prevalent in underserved areas, but the increased enrollment can have drawbacks. Because these plans often have more restrictive provider networks, potentially limiting access to care, and zero-premium plans consistently ranked lower than other plans in quality ratings, their growing prevalence raises concerns about the level of care beneficiaries receive. While zero-premium plans can improve initial affordability, they may not care needs of vulnerable populations, potentially exacerbating existing health disparities. The concerning juxtaposition of rapid growth, concentrated enrollment in vulnerable communities, and persistent lower quality ratings demands closer scrutiny by policymakers.

Key points

- Zero-premium MA plans are rapidly growing: Enrollment has surged significantly from 2019-2024, comprising a larger share of the MA market.
- Zero-premium plans are concentrated in vulnerable communities: These plans disproportionately enroll beneficiaries in counties with a higher proportion of racial/ethnic minority populations and prevalence in poverty and poorer health, potentially exacerbating existing inequities.
- Zero-premium plans may have lower quality: Lower star ratings raise concerns about the quality of care received by enrollees, particularly those in socioeconomically vulnerable areas.

## Introduction

Medicare beneficiaries can enroll in either Traditional Medicare or Medicare Advantage (MA) plans. After steadily growing over time, MA enrollment surpassed Traditional Medicine enrollment in 2024.^1,2^ The growth of MA reflects a trade-off: MA plans typically involve restrictions, such as limited provider networks and prior authorization,^3,4^ while offering potentially appealing flexibilities and supplemental benefits, such as prescription drug benefits or fitness programs.^5^

One potentially appealing benefit of MA plans is lower premiums. In Traditional Medicare, beneficiaries pay monthly Part B premiums ($174.70 in 2024), with additional income-related adjustments for higher earners.^6^ Meanwhile, MA beneficiaries also pay Part B premiums, but may avoid supplemental premiums altogether. Such “zero-premium” plans have become remarkably popular, currently serving more than 3/4 of MA beneficiaries.^1,7-10^ However, the perceived initial affordability of zero-premium MA plans may be overstated. While these plans can eliminate supplemental premiums, beneficiaries may incur substantial out-of-pocket spending through higher cost-sharing requirements, tiered prescription drug cost-sharing, and greater out-of-network expenses^11,12^ and may be offered fewer supplemental benefits as plans seek to offset the loss of premium revenue.^13,14^ Moreover, prior research has shown that lower-premium MA contracts often operate with narrower provider networks,^15-18^ which can limit access to specialty care, including high-quality clinicians and facilities.^14,19-22^ Together, these dynamics raise important questions about the reach and performance of zero-premium plans.

Despite the rapid growth in zero-premium MA enrollment, little is known about which communities are most likely to rely on these plans and how such plans differ in their structure and availability. These characteristics may be particularly consequential for socioeconomically vulnerable or clinically higher-need populations, who are more likely to be cost-sensitive and less able to navigate or absorb restrictions in coverage.

This study addressed these knowledge gaps by evaluating geographic variations in the enrollment trends in zero-premium MA plans, with a focus on county-level indicators of socioeconomic vulnerability and clinical need. We hypothesized that zero-premium plans would be disproportionately available in communities with greater needs, while plan quality would not differ significantly across premium types. By clarifying how zero-premium plans are distributed and adopted, findings from this work provide an empirical foundation for understanding whether their rapid growth is concentrated within particular communities and inform policy discussions on plan quality standards, equity in MA enrollment, and the potential need for greater oversight of enrollment incentives.

## Methods

### Data and sample

We constructed a county-level dataset spanning 2019-2024 by linking multiple public-use files from the Centers for Medicare and Medicaid Services (CMS) with county sociodemographic and health characteristics. Plan premium information was primarily obtained from the CMS Prescription Drug Plan Formulary, Pharmacy Network, and Pricing Information files and supplemented with premium data from the quarterly State/County/Contract/Plan (SPUF) enrollment files.^23-25^ Zero-premium plans were those with a total monthly beneficiary premium of $0, calculated as the sum of the Part C and, if applicable, Part D premiums. Data sources were linked using CMS contract, plan, and county identifiers. We restricted our analysis to plans that were most widely available to the public (health maintenance organization, HMO; health maintenance organization point of service, HMO-POS; preferred provider organizations, PPO) and excluded employer-sponsored and Supplemental Need Plans (including those for dual-eligible individuals) as well as plans with fewer than 10 enrollees. We also incorporated area-level data from the Centers for Disease Control and Prevention's latest 2022 Social Vulnerability Index (SVI), which provided a multidimensional composite measure of community socioeconomic vulnerability derived primarily from the American Community Survey (ACS) 5-year (2018-2022) estimates, and from the County Health Rankings.^26,27^ Counties were excluded from analysis if they had missing data for any of the 3 county-level characteristics: percent racial/ethnic minority, poverty, and poor health.

### Study measures

We examined 2 primary outcomes: (1) the number of zero-premium MA plans available in each county-year and (2) the county-level annual enrollment rate in zero-premium MA plans, calculated as the number of beneficiaries enrolled in zero-premium plans divided by the total number of MA beneficiaries (both zero-premium and non-zero-premium plans) in that county. For each county, we also examined 3 county-level characteristics reflecting population-level socioeconomic vulnerability or health need: (1) racial/ethnic minority percentage (proportion of residents identifying as racial or ethnic minorities), (2) poverty percentage (percentage of individuals living below 150% federal poverty line), and (3) poor health status (percentage of residents reporting poor or fair health). Counties were ranked from lowest to highest based on the values for each of these 3 characteristics and divided into quartiles (Q1 representing the least vulnerable or lowest need; Q4 representing the most vulnerable or highest need).

To understand the relative performance of each plan, we used CMS's Medicare Advantage Star Ratings. This 5-star rating system incorporates measures of clinical quality, member experience, and customer service, reflecting contract-level performance, and is widely used.^28^ While the rating system does not capture plan-specific variations within each contract, it provides an overall view as a general quality indicator. For our study, a rating of 1-3.5 stars was considered a low rating, while 5 stars represented the highest quality rating.

### Statistical analysis

We calculated annual counts and enrollment rates for zero- and non-zero-premium MA plans in US counties from 2019 to 2024. Choropleth maps were used to visualize the geographic distribution and gross changes in zero-premium enrollment during this period.

Using combined data from 2019-2024, we examined plan counts and enrollment rates for zero- and non-zero-premium MA plans, stratified by (1) county-level socioeconomic and health characteristics (racial/ethnic minority percentage, poverty percentage, and percentage reporting fair/poor health); (2) geographic region (Midwest, Northeast, South, West); (3) plan type (HMO, HMO-POS, PPO); and (4) star rating (1-3.5, 4-4.5, 5). Chi-squared tests were used to compare differences between these groups. Complementary multivariable generalized linear regression analyses were performed to examine the association between plan and county-level characteristics and zero-premium status. Within-county correlation was accounted for using cluster-robust standard errors at the county level.

Trends in zero-premium enrollment were assessed using Cochran-Armitage trend tests within quartiles of each county-level socioeconomic and health characteristic across the study period. Plan performance was assessed using year-specific star ratings, comparing the proportion of low-star rating (1-3.5) plans for zero-premium vs non-zero-premium plans, both overall and within counties stratified by socioeconomic and health characteristics for each county-year. All percentages were weighted by MA enrollment to reflect the variation of plan size.

All analyses were performed at the county-plan level using Python (version 3.9.19) and R (version 4.4.0). Two-sided statistical tests were used with a significance level of *P* < 0.05. This study was determined to be non-human subjects research by the UT Southwestern Human Research Protection Program.

## Results

**Figure 1.**
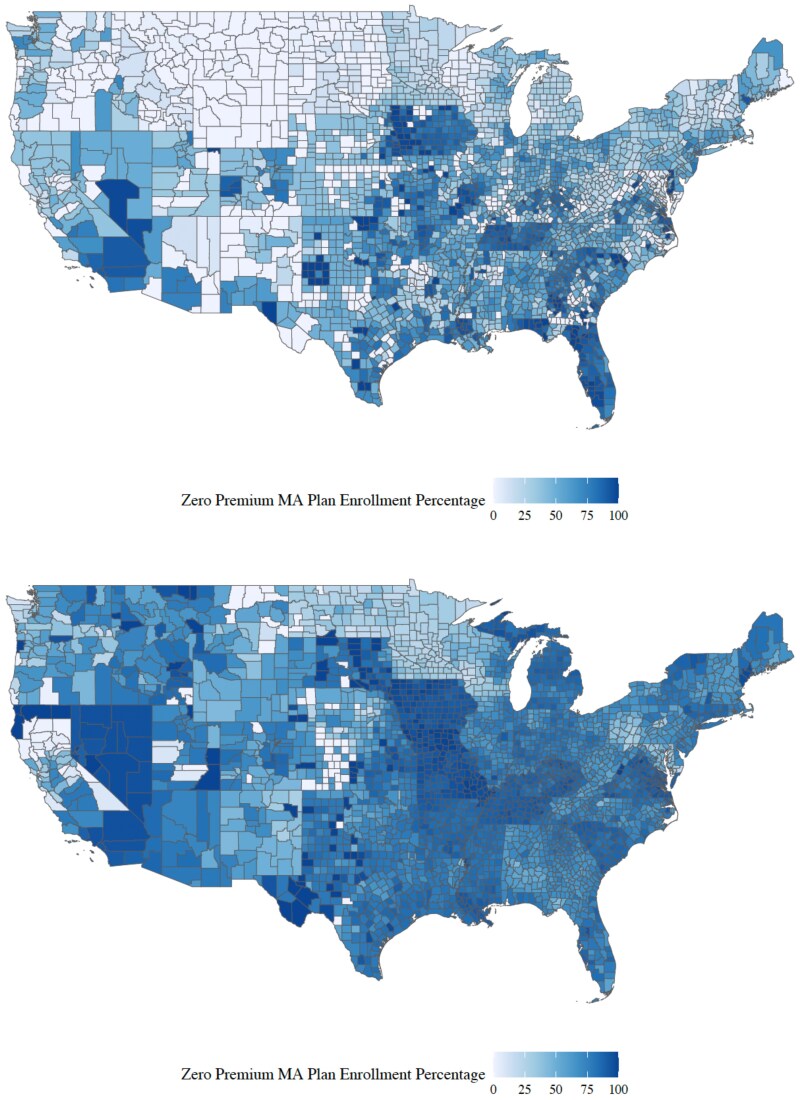
County-level zero-premium Medicare Advantage plan enrollment in the continental United States, 2019 vs 2024. Map limited to counties in the continental United States; Alaska, Hawaii, and other outlying islands of US territories are excluded. County-level data gaps were imputed using state averages.

From 2019 to 2024, our analytic dataset included a cumulative total of 166 406 county-year observations of zero-premium MA plans (59.7%) and 112 353 county-year observations of non-zero-premium MA plans (40.3%) across 2472 US counties (Table 1, eFigure S1). The availability of zero-premium MA plans increased substantially over time, rising from 12 381 plans (46.0% of all MA plans) with 9.12 million beneficiaries (59.8% of all MA beneficiaries) in 2019 to 45 255 plans (66.3%) with 18.76 million beneficiaries (74.3%) in 2024 (all *P*-trend < 0.001). In contrast, non-zero-premium plans and enrollment decreased proportionally, though there was modest absolute growth in the number of plans (from 14 521 to 23 001) and beneficiaries (from 6.13 to 6.51 million; all *P*-trend < 0.001), their proportions decreased. (Table 1, Figure 1)

**Table 1.** Trends in zero-premium and non-zero-premium MA plans and enrollment, 2019-2024

	2019	2020	2021	2022	2023	2024	Total	*P*
Plan, *n* (%)^a^								<0.001
Zero-premium plans	12 381 (46.02%)	16 718 (50.07%)	22 649 (55.25%)	30 898 (61.1%)	38 505 (65.65%)	45 255 (66.3%)	166 406 (59.7%)	
Non-zero-premium plans	14 521 (53.98%)	16 668 (49.93%)	18 343 (44.75%)	19 675 (38.9%)	20 145 (34.35%)	23 001 (33.7%)	112 353 (40.3%)	
Total	26 902	33 386	40 992	50 573	58 650	68 256	278 759	
Enrollment (by million), *n* (%)^b^								<0.001
Zero-premium plans	9.12 (59.8%)	10.76 (62.75%)	12.82 (66.14%)	14.92 (69.62%)	17.07 (72.47%)	18.76 (74.25%)	83.45 (68.4%)	
Non-zero-premium plans	6.13 (40.2%)	6.39 (37.25%)	6.56 (33.86%)	6.51 (30.38%)	6.49 (27.53%)	6.51 (25.75%)	38.59 (31.6%)	
Total	15.25	17.15	19.38	21.43	23.56	25.27	122.04	

Calculations concentrate on the plans most widely available to the public. We include HMO, HMO-POS, and PPO plans and excluded employer-sponsored and Supplemental Need Plans (including those for dual-eligible individuals) as well as plans with fewer than 10 beneficiaries.

^a^Calculated percentages represent the proportion of unweighted zero-premium vs non-zero-premium plan counts among all MA plans (zero premium and non-zero premium) within each year.

^b^Calculated percentages represent the proportion of zero-premium vs non-zero-premium plan beneficiaries among all MA plans (zero premium and non-zero premium) within each year.

Using combined data from 2019 to 2024, we observed that zero-premium plans exhibited the highest enrollment concentrations in the South (overall, 43%; annual range, 41.9%-44.3%) and West (overall, 23.3%; annual range, 22.1%-26.5%), while non-zero-premium plans were more evenly distributed across all regions (*P* < 0.001) (Table 2). Notably, zero-premium plan counts more than doubled in all regions during the study period, surpassing non-zero-premium plan counts by 2024. After adjusting for year and plan-level characteristics, supplementary multivariable analyses further confirmed higher odds of zero-premium MA plan availability in the West and South compared with the Northeast (*P* < 0.001) (Table 1, eTable S1, eTable S2).

**Table 2.** Characteristic summaries comparing zero-premium and non-zero-premium Medicare Advantage plan, 2019-2024 combined.

	Zero premium	Non-zero premium	*P*
*N*	166 406	112 353	
Region (Col %)^a^			< 0.001
Midwest	26 120 (18.27%)	26 120 (23.25%)	
Northeast	25 742 (15.47%)	22 030 (19.61%)	
South	71 564 (43.01%)	34 208 (30.45%)	
West	38 704 (23.26%)	29 994 (26.70%)	
Plan type (Col %)^a^			< 0.001
HMO	98 728 (59.33%)	53 236 (47.38%)	
HMO-POS	23 226 (13.96%)	20 560 (18.30%)	
PPO	44 452 (26.71%)	38 557 (34.32%)	
Plan star rating (Col %)^a^			< 0.001
1-3.5 star	37 918 (22.79%)	18 842 (16.77%)	
4-4.5 star	103 469 (62.18%)	70 125 (62.41%)	
5-star	18 154 (10.91%)	20 046 (17.84%)	
N/A	6866 (4.13%)	3341 (2.97%)	
County characteristics (Col %)^a,b^			
Minority Percentage^c^			< 0.001
Q1	10 274 (6.17%)	9670 (8.61%)	
Q2	20 508 (12.32%)	17 659 (15.72%)	
Q3	46 330 (27.84%)	35 806 (31.87%)	
Q4	89 294 (53.66%)	49 218 (43.81%)	
Poverty rate^d^			< 0.001
Q1	47 801 (28.73%)	43 556 (38.77%)	
Q2	58 585 (35.21%)	33 041 (29.41%)	
Q3	42 969 (25.82%)	25 314 (22.53%)	
Q4	17 051 (10.25%)	10 441 (9.29%)	
Self-reported fair or poor health status^e^			< 0.001
Q1	13 952 (8.38%)	18 161 (16.16%)	
Q2	35 601 (21.39%)	31 149 (27.72%)	
Q3	61 014 (36.67%)	35 933 (31.98%)	
Q4	55 840 (33.56%)	27 110 (24.13%)	

Data calculations concentrate on the plans most widely available to the public. We include HMO, HMO-POS, and PPO plans, excluding employer-sponsored plans, Supplemental Need Plans (including those for dual-eligible individuals), as well as plans with fewer than 10 beneficiaries. Abbreviations: HMO, health maintenance organizations; HMO-POS, health maintenance organization point-of-service; PPO, preferred provider organization.

^a^Absolute plan numbers were weighted by the enrollment rate of total MA enrollment across different levels of corresponding characteristics (ie, regions, plan types, plan star ratings, and county characteristics’ quartiles) from 2019 to 2024 for each premium plan type; percentages represent the enrollment rates with a sum of 100% for all characteristic columns under each premium plan type.

^b^Q4 represents counties ranking in the highest quartile/most vulnerable (top 25%) for each characteristic (racial/ethnic minority percentage, poverty rate, or poor/fair health status) among all counties in the study.

^c^The average percentile percentage minority [Hispanic or Latino (of any race); not Hispanic or Latino—Black and African American, American Indian and Alaska Native, Asian, Native Hawaiian and Other Pacific Islander, 2 or more races, other races] in the counties.

^d^The percentage of people below the 150% federal poverty line [data retrieved from the CDC's 2022 Social Vulnerability Index (SVI) Dataset].

^e^The percentage of adults reporting fair or poor health (age-adjusted) in the counties. The higher the value, the more adults in the suboptimal health status. [Data retrieved from 2022 County Health Ranking Dataset].

Plan types differed significantly, with zero-premium plans having more HMOs (59.3% vs 47.4%) and fewer PPOs (26.7% vs 34.3%) than non-zero-premium plans (*P* < 0.001). These patterns were generally consistent over time, although the proportion of HMOs among zero-premium MA plans reduced noticeably (Table 1, eTable S3). Supplementary multivariable results (eTable S2) confirmed these findings, showing that plan type remained a significant correlate of zero-premium status.

### Zero-premium plan enrollment by county socioeconomic or health needs

Across our study period, zero-premium MA plans exhibited complex patterns of concentration, with increasing presence in counties with higher percentage of racial/ethnic minority populations and individuals reporting poorer health status. Specifically, within the highest quartile for racial/ethnic minority percentage (Q4), 53.7% of all MA plans were zero-premium plans vs 43.8% non-zero premium (*P* < 0.001). Similarly, in counties within the highest quartile for self-reported fair or poor health status (Q4), 33.6% of plans were zero-premium compared to 24.1% non-zero premium (*P* < 0.001). In contrast, despite lower overall zero-premium concentration in counties with higher poverty rates, a modestly greater proportion of plans were zero-premium vs non-zero premium (10.3% vs 9.3%, *P* < 0.001) in counties within the highest poverty quartile (Q4). Results from supplementary multivariable regression analyses further support these descriptive results, demonstrating similar quartile-based disparities with statistical significance (Table 1, eTable S2).

**Figure 2.**
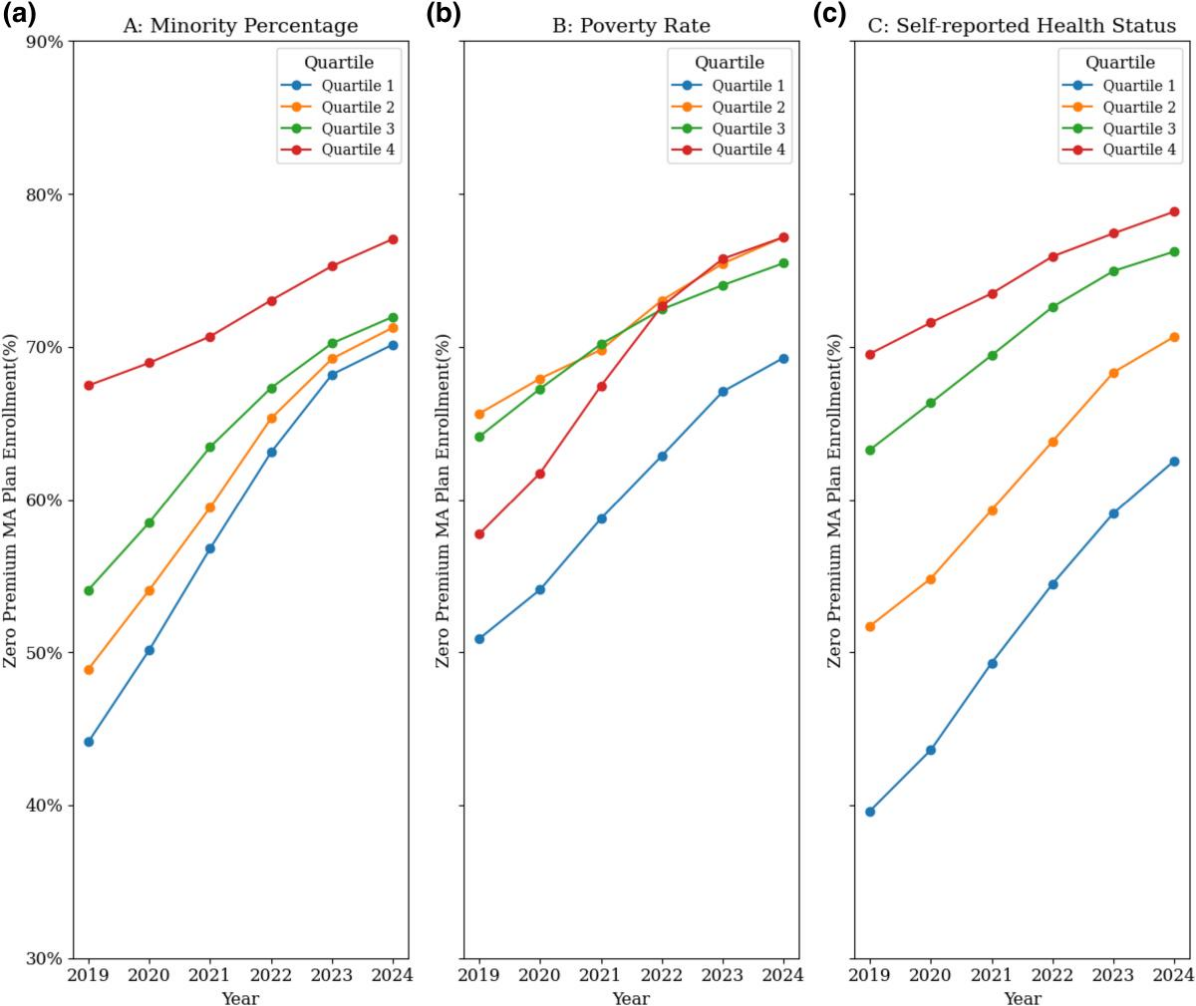
Trends in zero-premium plan enrollment by minority % (a), poverty rate (b), and health status (c) quartiles (2019-2024). Panel A: enrollment trends by county minority percentage (ranked in quartiles). Panel B: enrollment trends by county poverty rate (ranked in quartiles). Panel C: enrollment trends by county health statuses (ranked in quartiles). Counties were ranked into quartiles based on minority population percentage, poverty rate, and self-reported fair or poor health status. Q1 indicates counties in the least vulnerability quartile, and Q4 indicates those in the most vulnerable quartile. Values represent the percentage of MA beneficiaries enrolled in zero-premium plans within each quartile, relative to total MA enrollment in that quartile (eg, in 2019, 67% of MA beneficiaries in Q4-poverty counties were enrolled in zero-premium plans in Panel A). (D) The analysis includes HMO, HMO-POS, and PPO plans, excluding employer-sponsored plans, Special Needs Plans (SNPs, including those for dual-eligible beneficiaries), and plans with fewer than 10 enrollees, to focus on plans most widely available to the public.

Beyond the number of zero-premium plans offered, enrollment patterns were closely linked to county-level socioeconomic and health-related factors (Figure 2). Throughout the study period, counties in the highest quartile of racial/ethnic minority population (Q4) and the poorest self-reported health status (Q4) maintained consistently elevated zero-premium MA plan enrollment rates, increasing from 67.5% and 69.5% in 2019% to 77.1% and 78.8% in 2024, respectively (*P*-trend < 0.001) (Figures 2a and c). Most distinctively, counties with the highest poverty levels (Q4), despite lower initial zero-premium enrollment, showed the most rapid growth from 57.7% in 2019% to 77.2% in 2024 (*P* < 0.001), outpacing growth in all other poverty quartiles (Figure 2b).

### Zero-premium MA plan star rating

**Figure 3.**
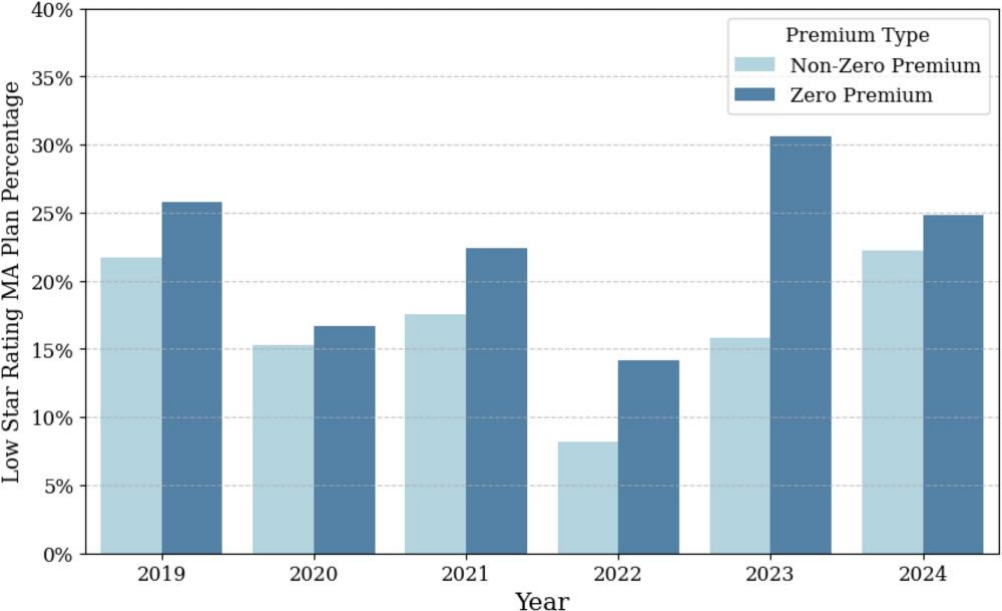
National trends in low star ratings by Medicare Advantage premium type (2019-2024). Low-rated plans are defined as those receiving 1-3.5 stars under the MA 5-star rating system. Percentages represent the enrollment-weighted share of low-rated plans within each premium category (zero premium or non-zero premium) by year. To focus on plans most broadly available to the public, the analysis includes HMO, HMO-POS, and PPO plans, excluding employer-sponsored plans, Special Needs Plans (SNPs, including those for dual-eligible beneficiaries), and plans with fewer than 10 enrollees.

Combining all years, nationally, zero-premium plans exhibited poorer performance compared to non-zero-premium plans: a significantly larger proportion had low star ratings (22.8% vs 16.8%), and a smaller proportion achieved 5-star ratings (10.9% vs 17.8%) (*P* < 0.001; Table 1). This disparity persisted across the study period. The gap in low-star-rating plans was greatest in 2023, when zero-premium plans had a 14.8% point higher share of low-rated contracts compared with non-zero-premium plans (30.7% vs 15.8%). Conversely, the difference in higher-quality plans peaked in 2022, with zero-premium plans showing a 17.0% point lower share of 5-star ratings relative to non-zero-premium plans (17.6% vs 34.6%, *P* = 0.016) (Figure 3, eTable S4A and B).

Throughout the study period, zero-premium MA plans consistently exhibited a higher proportion of low-star ratings compared to non-zero-premium plans across all county characteristics (percentage racial and ethnic minority population, poverty, and fair or poor health status). This disparity was particularly pronounced in later years (2021-2024), with zero-premium plans showing a substantially greater percentage of plans with low star ratings across all quartiles (eTable S4C).

## Discussion

In this national analysis, we observed a more than twofold increase over a 5-year period in the number and proportion of MA plans with zero premiums. Enrollment in these zero-premium plans was consistently the highest in counties with higher proportions of individuals from racial/ethnic minority groups, living in poverty, and reporting poorer health. Notably, compared to other MA plans, more of these zero-premium plans had lower quality ratings. These results are notable for several reasons.

First, our findings show an overall significant increase in zero-premium plan availability across US counties, with the number of plans nearly doubling from 2019 through 2024 in all regions. While an increased zero-premium plan enrollment rate was observed in almost all counties with available MA plans, heavier concentrations of zero-premium plan availability and national enrollment occurred in counties located in the South and West. Consistent with prior national reports, counties in the South and, to a lesser extent, the West have tended to be more socioeconomically vulnerable than those in the Midwest or Northeast, which may help explain the disproportionately high availability and enrollment in zero-premium MA plans in these regions.^29-32^ Nevertheless, the heterogeneous availability of zero-premium MA plans across US counties likely reflects a combination of market and regulatory factors, including county-level benchmark payments, plan cost structures, competitive dynamics, and network adequacy requirements.^33-35^ While our study was not designed to evaluate these drivers, future work using longitudinal data and linking market-level variables with plan bid and contracting data could better elucidate the determinants of geographic variation in plan availability.

Geographic disparities in availability and enrollment observed in our study align with a broader pattern in which zero-premium plans are disproportionately concentrated in counties with higher socioeconomic vulnerability and health needs, suggesting an important (and potentially concerning) interplay between supply-side (plans’ marketing strategies and offerings) and demand-side (beneficiaries’ financial constraints, socioeconomic pressures) factors.^36-38^ Plan strategies may include aggressive marketing (eg, unsolicited home visits and phone calls) and broker incentives.^5,14,39^ While these strategies are not inherently wrong, their transparency and responsiveness remain unknown (eg, whether seniors receive adequate information about plans’ network restrictions and potential out-of-pocket costs^40^). Evidence from prescription drug insurance indicates that older patients may place greater weight on upfront premium savings over expected out-of-pocket costs or other costly components of benefit design^39,41^ and some may mistakenly believe they are interacting with CMS when responding to marketing ads, only to subsequently enroll in plans with restrictive networks and unexpected out-of-pocket costs.^42^ These patterns could be particularly harmful for individuals facing financial constraints or other health-related social needs, underscoring the importance of transparency and informed decision-making about plan offerings.

Second, our finding that zero-premium MA plans have substantially higher enrollment in counties with more vulnerable sociodemographic profiles raises concerns about equitable access to care. Prior research has found that lower-premium MA plans are more often associated with narrower provider networks,^17,18,43,44^ while higher-premium plans have been linked to better plan quality and patient experience.^45,46^ In vulnerable communities (where health insurance literacy is often limited), beneficiaries may be more likely to select plans primarily based on premiums, with less awareness of trade-offs in network breadth, quality, or out-of-pocket costs.^36-38,47^ These choices are further reinforced by high plan “stickiness,”^48,49^ in which beneficiaries remain in the same plans for years, despite better or more affordable options, especially if they have only a limited understanding of their plan's network or cost-sharing implications in the absence of major health events. Compounding this, Medigap purchase is restricted by medical underwriting laws outside of the initial enrollment period, limiting the ability of older or sicker beneficiaries to switch back to traditional Medicare with supplemental coverage.^50^ Collectively, these dynamics may be associated with higher enrollment of vulnerable populations in plans that offer less robust provider networks and potentially lower quality of care, which could align with or contribute to existing health disparities.

Third, the concentration of zero-premium plans in vulnerable communities is further notable in light of the lower quality ratings observed among these plans. Our findings align with limited prior evidence suggesting that MA plans with lower star ratings are more often offered in counties with higher disease burdens and limited healthcare resources^51^ and that zero-premium plans, in particular, may be more frequently associated with lower quality measures than their premium-charging counterparts.^14,22,45^ Within this context, we observed a widening disparity in quality ratings, with a significantly higher proportion of low-star ratings among zero-premium plans compared to other plans, both nationally and within counties stratified by socioeconomic and health needs. These patterns are concerning, as they may reflect or reinforce existing health inequities.^47,51-53^

Taken together, these findings raise important questions about how the growth of zero-premium plans could affect health equity, particularly in counties with high disease burdens but limited access to healthcare resources. Future research should evaluate whether these plans’ benefits, provider networks, and cost-sharing requirements adequately meet the needs of beneficiaries in such settings and assess their impact on health outcomes using longitudinal patient-level data across diverse populations. Additional priorities include examining financial incentives driving broker recommendations and marketing practices, as well as understanding beneficiary decision-making in the context of limited health insurance literacy and high plan inertia. Such work should explore how beneficiaries weigh the appeal of zero additional monthly premiums and supplemental benefits against potential barriers such as narrower provider networks or prior authorization requirements.^16,17,54^

From a policy perspective, closer surveillance at both federal and state levels is warranted to ensure that initial affordability does not come at the expense of network adequacy, care quality, patient financial hardship, or worse health outcomes. Enhanced transparency on provider network breadth and composition should be paired with quality measures that more directly reflect management of patient-level social and clinical risks. To help ensure that zero-premium plans deliver on their promise of value without exacerbating existing disparities, policymakers could also prioritize efforts to strengthen regulatory oversight of star ratings to better account for social risk factors, expand targeted beneficiary education programs, and evaluate broker incentive structures. Such measures would promote informed choice, equitable access, and sustained quality of care for vulnerable populations as these plans continue to expand.

This study has limitations worth noting. First, our reliance on county-level data may mask important within-county heterogeneity in social determinants of health and plan characteristics, limiting our ability to capture individual-level variations in enrollment decisions, healthcare needs, or outcomes. The SVI provides valuable insights into the socioeconomic context of the areas where enrollees reside, but it does not directly reflect the demographics or socioeconomic status of the enrollees themselves. Future research should investigate the individual-level characteristics of zero-premium plan enrollees to confirm whether their demographics align with the trends suggested by our area-level analysis. Second, excluding plans without valid premium information or prescription drug coverage may affect the generalizability of the findings. However, this is unlikely to substantially alter our conclusions, as 97% of Medicare Advantage beneficiaries were also enrolled in plans with prescription drug coverage in 2024.^3^ Third, our study period encompassed the COVID-19 pandemic, which may represent a potential unmeasured confounder. During this time, beneficiaries’ preferences often shifted toward plan affordability and the expanded supplemental benefits offered by MA plans^55,56^ (amid social isolation and financial hardship) likely making zero-premium options particularly attractive and sustaining, if not accelerating, their growth. While we lack direct data to quantify the exact shift attributable to the pandemic, the appeal of zero-premium plans predates this period, and enrollment continued to expand after 2022. The pandemic likely amplified, rather than initiated, this growth trajectory. Fourth, while star ratings are widely used to assess MA plan quality, questions have been raised about their sensitivity to social risk factors and their ability to fully capture the experiences of minority populations.^57^

Furthermore, due to data limitations, star ratings were estimated at the contract level, which may mask heterogeneity in plan performance across service areas and introduce measurement error compared to plan-level assessments.^58^ In addition, the star rating system itself has recognized limitations, including potential inflation of “high” ratings due to quality bonus incentives,^22,53,58,59^ reliance on plan-reported measures, and incomplete adjustment for social and clinical risk factors, which may limit its ability to capture the experiences of vulnerable populations.^45,52,57,60^ Nonetheless, given their widespread use in policy and beneficiary decision-making, star ratings remain a relevant—though imperfect—indicator of plan quality. Our focus on low-star ratings (1-3.5 stars) provides a more conservative signal of potential quality concerns, with the likelihood that their prevalence is underestimated under current rating practices. As regulators continue to refine the methodology for calculating star ratings, we are optimistic that future research will benefit from a more accurate and reliable quality metric, improving its value as a reference for both policy evaluation and beneficiary choice.^58,61,62^

Finally, our study was designed as a descriptive analysis with an economic lens, relying on aggregate county-level data and lacking adjustment for unmeasured individual- and plan-level factors. As such, the findings should not be interpreted as establishing causal relationships. Notwithstanding, our study provides important evidence of a relationship between zero-premium plan availability, socioeconomic factors, and plan quality, underscoring the need for further investigation and enhanced quality oversight to ensure vulnerable populations have access to high-quality care.

## Conclusion

Our study highlights that enrollment in zero-premium MA plans grew substantially over time, with a concentration of lower quality plans in counties with greater socioeconomic vulnerability or health needs. Future studies should examine the implications of these trends and their impact on health outcomes in historically underserved populations.

## Contribution statement

C.J. and L.H. had full access to all the data in the study and take responsibility for the integrity of the data and the accuracy of the data analysis. Concept and design: C.J., J.M.L., C.A.L. Acquisition, analysis, or interpretation of data: all authors. Drafting of the manuscript: all authors. Critical review of the manuscript for important intellectual content: all authors. Statistical analysis: C.AL, L.H. Supervision: J.M.L., C.J.

## Supplementary Material

qxaf177_Supplementary_Data
