# Supplementary material for: Zero-premium Medicare Advantage plans: trends in areas with socioeconomic vulnerability and health needs
Source: Health Aff Sch. 2025 Sep 19;3(9):qxaf177. doi: 10.1093/haschl/qxaf177 (PMC12449130; doi:10.1093/haschl/qxaf177)
Supplement: qxaf177_Supplementary_Data [file qxaf177_supplementary_data.zip › Supplement_083025.pdf]

eFigure 1. Study Flowchart of County-Plan Selection in Medicare Advantage, 2019–2024

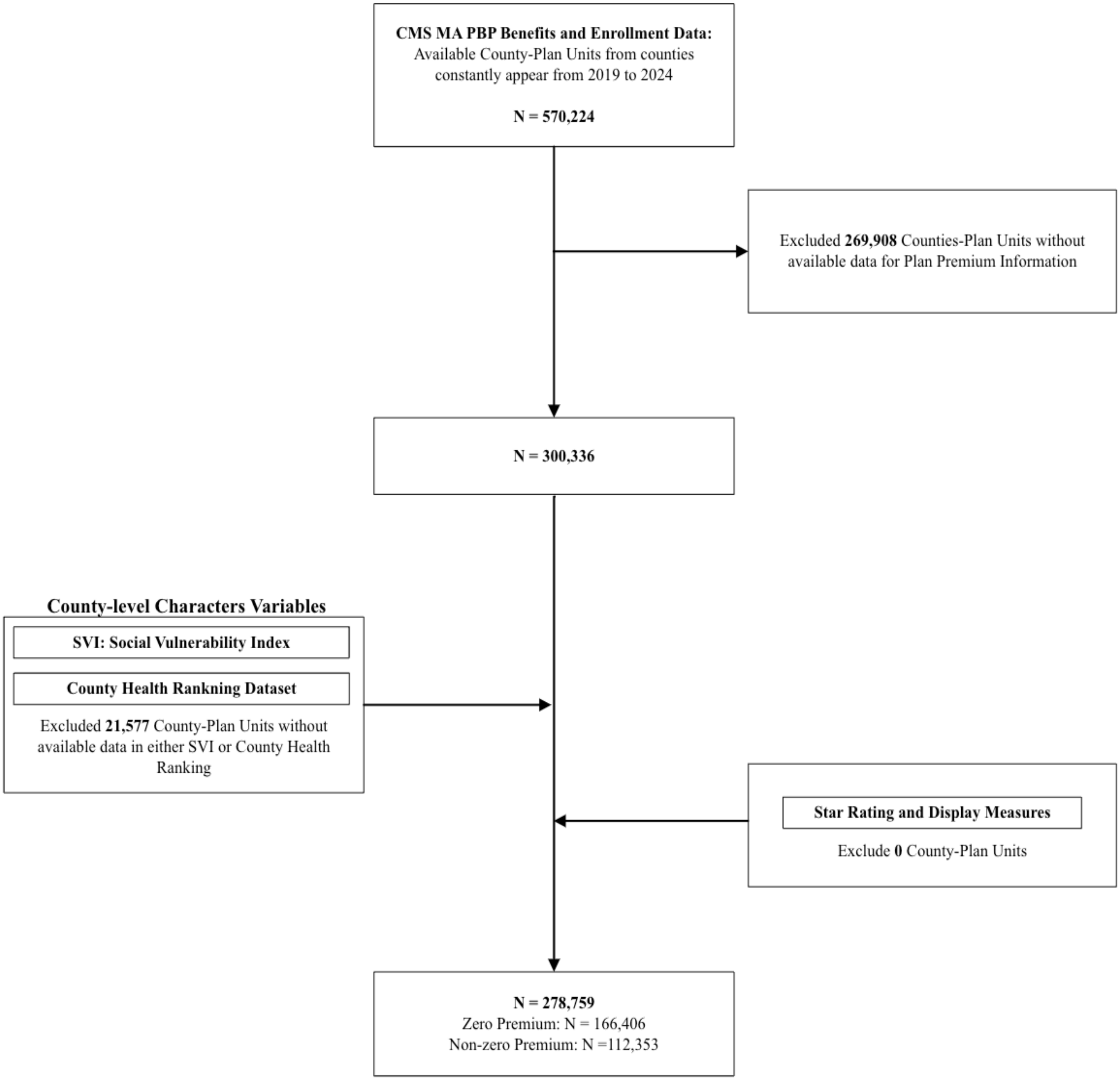

eTable 1. Geographic Distribution Trends in Medicare Advantage Plans by Premium Type, 2019–2024

Year	**Region**	Zero-Premium %	Non-Zero Premium %
2019	Midwest	2883 (15.86%)	4213 (23.60%)
	Northeast	2644 (14.54%)	4007 (22.45%)
	South	7836 (43.10%)	4780 (26.78%)
	West	4818 (26.50%)	4848 (27.17%)
2020	Midwest	3610 (16.83%)	4441 (23.87%)
	Northeast	3208 (14.95%)	3895 (20.94%)
	South	9486 (44.21%)	4944 (26.58%)
	West	5153 (24.01%)	5322 (28.61%)
2021	Midwest	4501 (17.61%)	4525 (23.68%)
	Northeast	3968 (15.52%)	3689 (19.30%)
	South	11331 (44.33%)	5412 (28.32%)
	West	5761 (22.54%)	5485 (28.70%)
2022	Midwest	5568 (18.71%)	4347 (22.93%)
	Northeast	4728 (15.89%)	3437 (18.13%)
	South	12869 (43.25%)	5956 (31.41%)
	West	6587 (22.14%)	5219 (27.53%)
2023	Midwest	6534 (19.19%)	4265 (22.58%)
	Northeast	5333 (15.66%)	3435 (18.18%)
	South	14370 (42.21%)	6480 (34.31%)
	West	7808 (22.93%)	4709 (24.93%)
2024	Midwest	7299 (19.51%)	4330 (22.86%)
	Northeast	5861 (15.67%)	3568 (18.83%)
	South	15671 (41.89%)	6636 (35.03%)
	West	8578 (22.93%)	4411 (23.28%)

Footnote:
Absolute plan counts were weighted by Medicare Advantage (MA) enrollment proportions across each level of the corresponding region from 2019 to 2024, separately for zero-premium and non-zero premium plan categories. Percentages reflect the share of enrollment within each characteristic column, such that percentages sum to 100% within each premium category for a given year.

eTable 2. Adjusted Association Between Plan/County Characteristics and Zero-Premium, 2019–2024

Plan-level Characteristics	Adjusted Odds Ratio[a] (95% CI)	P value
**Year*	1.24 (1.23 – 1.24)	p < 0.001
**Plan Type: [Ref =HMO]**		
HMO/POS	0.44 (0.42-0.46)	p < 0.001
Local PPO	0.47 (0.45-0.48)	p < 0.001
**Region: [Ref= Northeast]**		
Midwest	0.81 (0.73-0.90)	p < 0.001
South	1.60 (1.51-1.70)	p < 0.001
West	1.19 (1.12-1.27)	p < 0.001
**County-level Characteristics**	**Estimates[b] (95% CI)**	**P value**
**Year*	0.052 (0.05 – 0.053)	p < 0.001
Minority Percentage	0.047 (0.017 – 0.077)	p = 0.020
Poverty Rate	-0.056 (-0.104 – -0.009)	p = 0.002
Self-Reported Fair or Poor Health Status	0.504 (0.189 – 0.819)	p = 0.002

Footnote: * *Year* indicates per-year changes in odds or rates over the 2019–2024 period.

a. Generalized linear model (GLM) with binomial family and logit link was used to model the relationship between plan characteristics and the odds of having a zero-premium MA plan, adjusting for year, region, and county-level socioeconomic covariates where the plan belongs with. Cluster-robust standard errors were calculated at the county level using the sandwich variance estimator.

Linear mixed-effects regression was used to model the relationship between county-level characteristics and zero-premium MA plan enrollment rate, adjusting for year and region, with random intercepts for counties. For each county-level covariate, a one-quartile difference corresponds to moving from one quartile to the next in the distribution of that characteristic.

eTable 3. Trends in Medicare Advantage Plan Type by Premium Category, 2019–2024

Year	**Plan Type**	Zero-Premium %	Non-Zero Premium %
2019	HMO	14799 (81.40%)	10502 (58.84%)
	HMO/POS	412 (2.26%)	1749 (9.80%)
	PPO	2971 (16.34%)	5598 (31.36%)
2020	HMO	15594 (72.67%)	10302 (55.38%)
	HMO/POS	1679 (7.83%)	2338 (12.57%)
	PPO	4184 (19.50%)	5962 (32.05%)
2021	HMO	17232 (67.41%)	10215 (53.45%)
	HMO/POS	2191 (8.57%)	2543 (13.31%)
	PPO	6138 (24.01%)	6353 (33.24%)
2022	HMO	18595 (62.50%)	10043 (52.97%)
	HMO/POS	2821 (9.48%)	2330 (12.29%)
	PPO	8337 (28.02%)	6586 (34.74%)
2023	HMO	15856 (46.57%)	6419 (33.99%)
	HMO/POS	7620 (22.38%)	5806 (30.74%)
	PPO	10569 (31.05%)	6663 (35.28%)
2024	HMO	16653 (44.52%)	5756 (30.38%)
	HMO/POS	8504 (22.73%)	5794 (30.58%)
	PPO	12253 (32.75%)	7396 (39.04%)

Footnote: HMO= Health Maintenance Organizations; HMO/POS= Health Maintenance Organizations/Point-of-service; PPO= Preferred Provider Organization.

Absolute plan counts were weighted by Medicare Advantage enrollment proportions across each level of the corresponding plan type from 2019 to 2024, separately for zero-premium and non-zero premium plan categories. Percentages reflect the share of enrollment within each characteristic column, such that percentages sum to 100% within each premium category for a given year.

eTable 4A. National Trends in Medicare Advantage Low-Star Ratings by Premium Type, 2019–2024

Premium Type (%)	2019	2020	2021	2022	2023	2024
Non-Zero Premium	21.7	15.33	17.57	8.22	15.84	22.22
Zero Premium	25.78	16.65	22.4	14.2	30.65	24.79

Footnote:
Low-rated plans are defined as those receiving 1-3.5 stars under the MA 5-star rating system. Percentages represent the enrollment-weighted share of low-rated plans within each premium category (zero- or non-zero-premium) by year.

eTable 4B. Trends in Medicare Advantage Star Ratings by Premium Type, 2019–2024

Year	**Star Rating**	Zero-Premium %	Non-Zero Premium %
2019	1-3.5 star	4687 (25.78%)	3873 (21.70%)
	4-4.5 star	11506 (63.29%)	11581 (64.89%)
	5-star	1456 (8.01%)	1943 (10.89%)
	N/A	532 (2.92%)	451 (2.53%)
2020	1-3.5 star	3572 (16.65%)	2852 (15.33%)
	4-4.5 star	14835 (69.14%)	12819 (68.91%)
	5-star	2009 (9.36%)	2342 (12.59%)
	N/A	1041 (4.85%)	589 (3.17%)
2021	1-3.5 star	5724 (22.40%)	3358 (17.57%)
	4-4.5 star	16113 (63.04%)	11844 (61.97%)
	5-star	1568 (6.13%)	2609 (13.65%)
	N/A	2156 (8.43%)	1300 (6.80%)
2022	1-3.5 star	4225 (14.20%)	1558 (8.22%)
	4-4.5 star	19243 (64.68%)	10474 (55.24%)
	5-star	5240 (17.61%)	6567 (34.64%)
	N/A	1045 (3.51%)	360 (1.90%)
2023	1-3.5 star	10435 (30.65%)	2993 (15.84%)
	4-4.5 star	17842 (52.41%)	10874 (57.57%)
	5-star	4933 (14.49%)	4714 (24.96%)
	N/A	835 (2.45%)	308 (1.63%)
2024	1-3.5 star	9274 (24.79%)	4209 (22.22%)
	4-4.5 star	23930 (63.97%)	12533 (66.15%)
	5-star	2949 (7.88%)	1871 (9.88%)
	N/A	1256 (3.36%)	332 (1.75%)

Footnote:
Absolute plan counts were weighted by Medicare Advantage enrollment proportions across each level of the corresponding plan star rating from 2019 to 2024, separately for zero-premium and non-zero premium plan categories. Percentages reflect the share of enrollment within each characteristic column, such that percentages sum to 100% within each premium category for a given year.

eTable 4C. Trends in Medicare Advantage Low-Star Ratings by County Quartiles and Premium Type, 2019 – 2024

County Characteristics	Premium Type	2019	2020	2021	2022	2023	2024
**Minority Percentage[a]**							
Q1	Zero Premium	37.29%	18.67%	23.10%	8.66%	21.89%	20.82%
	Non-Zero Premium	8.69%	7.22%	7.61%	1.92%	7.18%	10.78%
Q2	Zero Premium	32.30%	12.88%	19.61%	9.19%	23.39%	20.70%
	Non-Zero Premium	12.50%	8.34%	11.20%	4.12%	10.95%	17.06%
Q3	Zero Premium	26.16%	17.35%	21.64%	11.68%	31.00%	20.64%
	Non-Zero Premium	17.94%	13.86%	14.48%	4.90%	14.09%	22.26%
Q4	Zero Premium	23.56%	16.88%	23.32%	17.43%	33.42%	28.68%
	Non-Zero Premium	30.80%	20.72%	24.06%	13.20%	20.35%	26.18%
**Poverty Rate[b]**							
Q1	Zero Premium	24.25%	14.60%	18.86%	13.17%	28.35%	26.60%
	Non-Zero Premium	17.39%	11.72%	13.97%	6.94%	16.02%	25.83%
Q2	Zero Premium	25.37%	16.03%	21.72%	12.82%	33.08%	24.59%
	Non-Zero Premium	22.07%	14.85%	19.35%	8.80%	16.89%	23.32%
Q3	Zero Premium	23.41%	16.66%	23.44%	14.44%	28.85%	22.90%
	Non-Zero Premium	22.19%	18.87%	18.64%	7.97%	13.72%	16.41%
Q4	Zero Premium	40.25%	25.41%	32.11%	20.88%	33.35%	24.87%
	Non-Zero Premium	38.12%	23.82%	24.35%	12.35%	17.13%	18.84%
**Self-reported Fair or Poor Health Status[c]**							
Q1	Zero Premium	28.21%	16.38%	16.75%	14.09%	31.26%	25.97%
	Non-Zero Premium	16.33%	9.73%	10.50%	5.59%	15.42%	25.84%
Q2	Zero Premium	29.01%	15.06%	20.77%	12.91%	29.76%	29.43%
	Non-Zero Premium	18.25%	12.05%	14.66%	7.02%	15.75%	27.61%
Q3	Zero Premium	29.54%	17.66%	22.75%	13.85%	33.73%	22.79%
	Non-Zero Premium	25.22%	18.63%	20.74%	8.97%	16.79%	20.32%
Q4	Zero Premium	19.36%	16.57%	24.35%	15.43%	27.69%	23.54%
	Non-Zero Premium	24.96%	18.81%	21.56%	10.33%	15.02%	16.75%

Footnote:

Percentages indicate the share of enrollment in plans with 1–3.5 star ratings, calculated within each premium type across counties in a given characteristic quartile. Q4 denotes counties in the most disadvantaged quartile (top 25%) for minority population percentage, poverty rate, or poor/fair health status.

a. The average percentile percentage minority (Hispanic or Latino (of any race); Not Hispanic or Latino - Black and African American, American Indian and Alaska Native, Asian, Native Hawaiian and Other Pacific Islander, Two or More Races, Other Races) in the counties.
b. The percentage of people below the 150% federal poverty line. [Data retrieved from the CDC's 2022 Social Vulnerability Index (SVI) Dataset]
c. The percentage of adults reporting fair or poor health (age-adjusted) in the counties. The higher the value, the more adults in the suboptimal health status. [Data retrieved from 2022 County Health Ranking Dataset]
